# Building an automated, machine learning-enabled platform for predicting post-operative complications

**DOI:** 10.1088/1361-6579/acb4db

**Published:** 2023-02-09

**Authors:** Jeremy A Balch, Matthew M Ruppert, Benjamin Shickel, Tezcan Ozrazgat-Baslanti, Patrick J Tighe, Philip A Efron, Gilbert R Upchurch, Parisa Rashidi, Azra Bihorac, Tyler J Loftus

**Affiliations:** 1 Intelligent Critical Care Center, University of Florida, Gainesville, FL, United States of America; 2 Department of Surgery, University of Florida, Gainesville, Florida, United States of America; 3 Department of Medicine, University of Florida, Gainesville, Florida, United States of America; 4 Department of Anesthesiology, University of Florida, Gainesville, Florida, United States of America; 5 Department of Biomedical Engineering, University of Florida, Gainesville, Florida, United States of America

**Keywords:** clinical decision support, artificial intelligence, machine learning, surgery, post operative complications

## Abstract

*Objective*. In 2019, the University of Florida College of Medicine launched the *MySurgeryRisk* algorithm to predict eight major post-operative complications using automatically extracted data from the electronic health record. *Approach*. This project was developed in parallel with our Intelligent Critical Care Center and represents a culmination of efforts to build an efficient and accurate model for data processing and predictive analytics. *Main Results and Significance*. This paper discusses how our model was constructed and improved upon. We highlight the consolidation of the database, processing of fixed and time-series physiologic measurements, development and training of predictive models, and expansion of those models into different aspects of patient assessment and treatment. We end by discussing future directions of the model.

## Introduction

13% of patients undergoing surgery will experience post-operative complications, with 1% resulting in mortality (Dencker *et al* 2021). With 48 million inpatient surgeries in the United States, this translates to over 6 million complications and 480 000 deaths (Stanford Health Care: Surgery Statistics: @StanfordHealth 2022). The University of Florida College of Medicine launched the *MySurgeryRisk* algorithm in 2019 to predict eight major post-operative complications (Bihorac *et al* 2019). The program represents one of the first attempts at integrating electronic health records with machine learning-enabled predictive tools in the field of surgery. In this article, we trace the evolution of the *MySurgeryRisk* algorithm from its inception to its proposed integration into clinical practice.

Prediction models have been used in the field of surgery for decades. The American Society of Anesthesiologists (ASA) classification has its roots in the 1940s while more objective and complex scoring systems came later, including the Physiological and Operative Severity Score for the Enumeration of Mortality and Morbidity (POSSUM, 1991), the National Surgical Quality Improvement Program (NSQIP) Surgical Risk Calculator (2013), the Surgical Outcome Risk Tool (SORT, 2014), and the Emergency Surgery Score (ESS, 2016) (Copeland *et al* 1991, Hyder* et al* 2016, Protopapa *et al* 2014, Sangji *et a*l 2016, Hashimoto *et al* 2021). These models are constructed with presumed linear, additive relationships between variables, meaning that static weights of morbidity and mortality are ascribed to each risk factor, regardless of how those risk factors interact with each other. Machine learning technologies are better suited to interpret the complex interactions between individual risk factors.

When it comes to pre-operative risk stratification, the Predictive OpTimal Trees in Emergency Surgery Risk (POTTER) used optimal classification trees to provide more accurate predictions than ASA or ESS (Hyder *et al* 2016, Hashimoto *et al* 2021). However, this model was developed on the pre-processed NSQIP dataset. Corey *et al* built Pythia, a machine learning tool with their own institutional data to develop a calculator for risk prediction and were working on further electronic health record (EHR) integration using a linear model (Corey *et al* 2018, Hashimoto *et al* 2021). While a recent review of surgical risk prediction models demonstrated a high-level of performance, they were limited to predicting overall mortality or composite morbidity (Reilly *et al* 2021). In order provide a real-time tool for predicting multiple possible complications, trained on locally available data with nonlinearity, our group sought to create a prediction tool that merges the EHR with prediction algorithms.

The University of Florida Intelligent Critical Care Center embodies the goal of bringing together experts in Medicine, Informatics, Computer Science, Engineering, Healthy Policy, and the Fine Arts to develop artificial intelligence tools to advance patient health (Intelligent Critical Care Center IC3 2022). Our group has had over 110 publications since its inception. The University of Florida currently houses one of the most powerful and efficient university-owned supercomputers in the world as part of our collaboration with NVIDIA (ai.ufl.edu-AI 2022). Our goal is to leverage the myriad resources and areas of expertise at the University of Florida to integrate the diverse inputs from electronic health records to an explainable tool for clinicians. This is essential for the multi-disciplinary nature of patient care.

To this end, we developed the *MySurgeryRisk* calculator and continue to build upon this model with the goal of integrating artificial intelligence and clinical practice to improve surgical outcomes.

## Database

The original dataset contained information from 51 457 patients undergoing major inpatient surgery at the University of Florida between January 1, 2000 and November 30, 2010. Using the University of Florida Health (UFH) Integrated Data Repository (IDR) as an honest broker, we assembled information on patient demographics, diagnoses, procedures, outcomes, comprehensive total charges, insurance status, and ICD-9-CM codes for diagnoses and procedures (Bihorac *et al* 2013). We aggregated data from the electronic health record with data from the Florida Bureau of Vital Statistics, the Social Security Death Index, the US Renal Data System, and US Census data, linking on social security number through the IDR for the death index and the first four digits of home-address zip for area development indices. Additional laboratory, pharmacy, and blood bank database were also merged. During this time, Centricity Perioperative Management and Anesthesia (General Electric Healthcare Inc.) were incorporated for relevant intraoperative variables (Bihorac *et al* 2013). The resulting de-identified perioperative database was called DECLARE.

DECLARE was updated to IDEALIST in 2021, with an additional 43,943 patients undergoing 52 529 surgeries between June 1, 2014 and September 20, 2020 following re-standardization of our database with the introduction of EPIC EHR to the University of Florida in 2011 along with the use of ICD-10-CM and CPT codes (Datta *et al* 2020). As of fall 2022, clinical text has been incorporated into our data set. A full list of variables can be found in Supplementary table 1.

## Data processing and physiologic time series

Electronic health record data requires extensive processing. Variables were securely transferred from the EHR to our processors using Secure Sockets Layer (SSL) protocol encryption with additional layers of protection to prevent SQL injections and other malicious attacks (Feng *et al* 2017). The data collects batches records into JSON string and writes to multiple serves in our processing cluster using Apache Kafka (Feng *et al* 2017). Outlier detection and removal was performed on laboratory results by replacing the top and bottom 1% of data using random uniform values generated from the 95% to 99.5% and 0.5% to % percentiles, respectively (Adhikari *et al* 2019). Categorical and nominal variables with multiple levels (such as surgeon’s identities and zip codes) were converted to numerical values by calculating the log of the ratio of the prevalence of a particular variable among cases with and without a complication.

A large portion of our models are dedicated to processing physiologic time series. Our group imputed time-series data using minimum, maximum, average, and short- and long-term variability measurements (Adhikari *et al* 2019). Short- and long-term variability were computed using the base and residual signals through use of a convolution filter (Saria *et al* 2010). Long-term variability is defined as the standard deviation of the base signal and the short-term variability is defined as the standard deviation of the residual signal (Saria *et al* 2010). Time-series were then passed into a neural network with gated recurrent units that has previously demonstrated optimal performance for sequentially ordered temporal data (Cho *et al* 2014). The model internally and continuously updated its parameters based on both current inputs and those from previous time steps, detecting patterns across the duration of operation or stay in the ICU. Longer-range temporal relationships are handled by GRUs.

Given the large dimensionality of our data set, two data reduction techniques were tested: Least Absolute Shrinkage and Selection Operator (LASSO) and Principal Component Analysis (PCA) (Thottakkara *et al* 2016). Data pre-processing was able to significantly reduce computational time in logistic regression and GAM, though SVM remained computationally intensive, requiring 2–3 h for one simulation. LASSO did not affect predictive performance, but PCA improved predictive power.

## Initial analytic models

Prior to 2012, there was limited work on machine learning at the University of Florida, and few published studies for clinical medicine in general. One of the first uses of this technology by our group to was to predict consultation of the Acute Pain Service (Tighe *et al* 2012). In a retrospective cohort trial, we developed supervised machine learning to develop classifiers to predict which procedures will require acute pain service consultation. Fifteen types of machine learning classification schemes were tested and compared using area under the curve (AUC), accuracy, sensitivity, specificity, and computational requirements. The model was optimized using ensemble development (averaging probability estimates of select machine learning techniques to generate a single output) and dimensionality reduction (identification of only the inputs that most influence the output). The Bayesian methods gave the highest AUC (0.87, 95% CI 0.84–0.89) for predicting acute pain service consults and six separate attributes were noted to contribute most highly to the outcome. Ensemble methods did not improve processing time or model accuracy. This result was likely due to highly patterned care protocols and provided early insight on the need to incorporate additional context on clinical processes and outcome measures in machine learning experiments. Key components of each our models are highlighted in supplementary tables 2 and 3.

The next iteration was a similar comparison of prediction models for postoperative sepsis and acute kidney injury. Published in 2016, Thottakkara *et al* examined logistic regression, generalized additive models, Naïve Bayes, and support vector machines (Thottakkara *et al* 2016). While logistic regression is commonplace in medical literature, it is a linear predictive model that will proportionally increase or decrease along with the predictor variables. Generalized additive models (GAM) can estimate non-linear functions with an interpretable solution and support vector machines (SVM) create a ‘hyperplane’ to separate data points. GAM models were best suited for building risk prediction models given their high discrimination and ability to account for the non-linearity of continuous variables. These models yielded AUC characteristics between 0.79 (Naïve Bayesian, 95% CI 0.79–0.80) and 0.86 (GAM, CI 0.85–0.86) for acute kidney injury and between 0.76 (SVM, 95% CI 0.74–0.78) and 0.91 (GAM, 95% CI 0.90–0.92) for severe sepsis.

**Figure 1.**
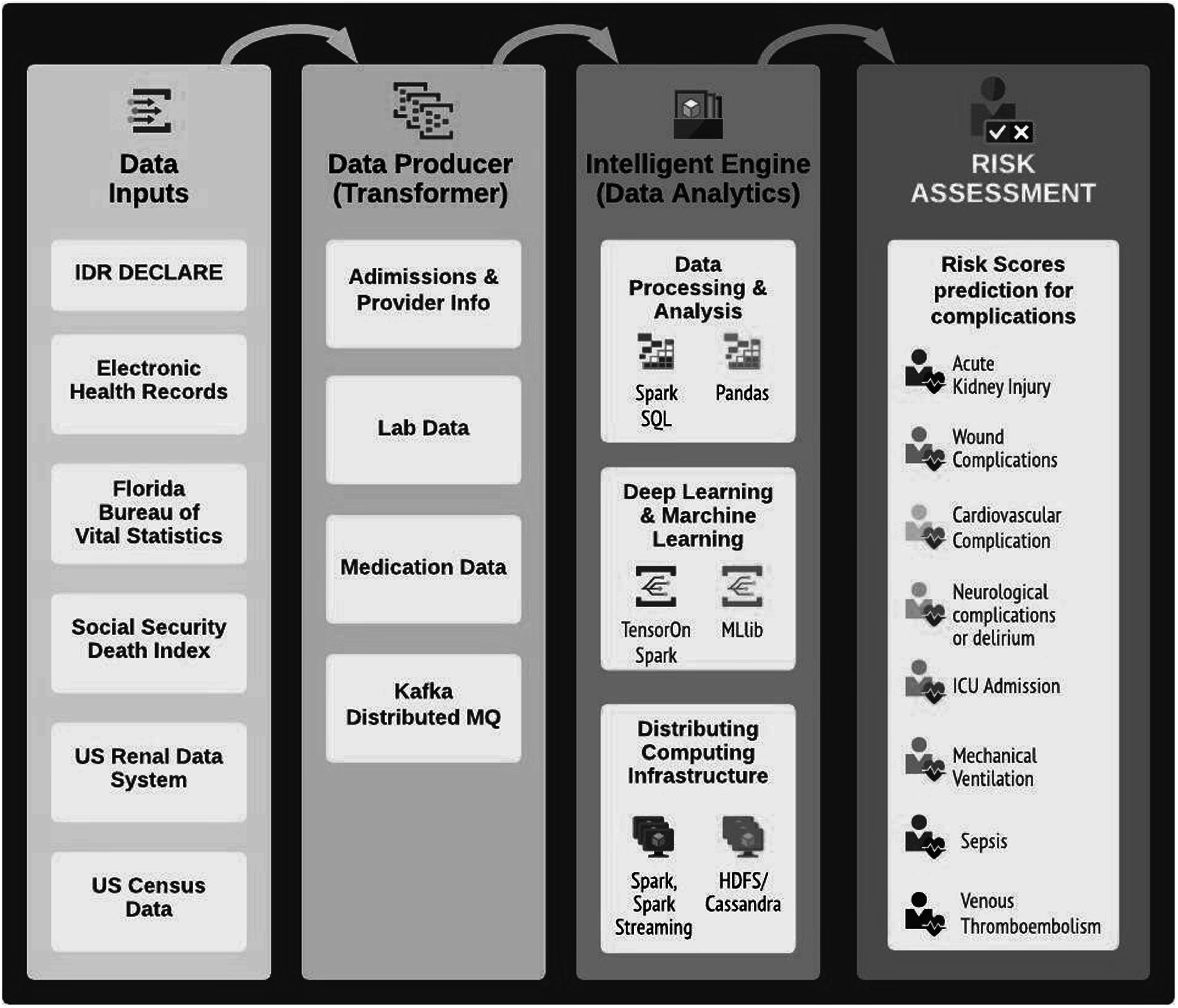
Original theoretical architecture described by Feng *et al* (2017). The system is separated into three components, taking inputs from multiple data sources, transforming the data for analysis by a several proposed methods to develop a risk assessment for eight post operative complications.

**Figure 2.**
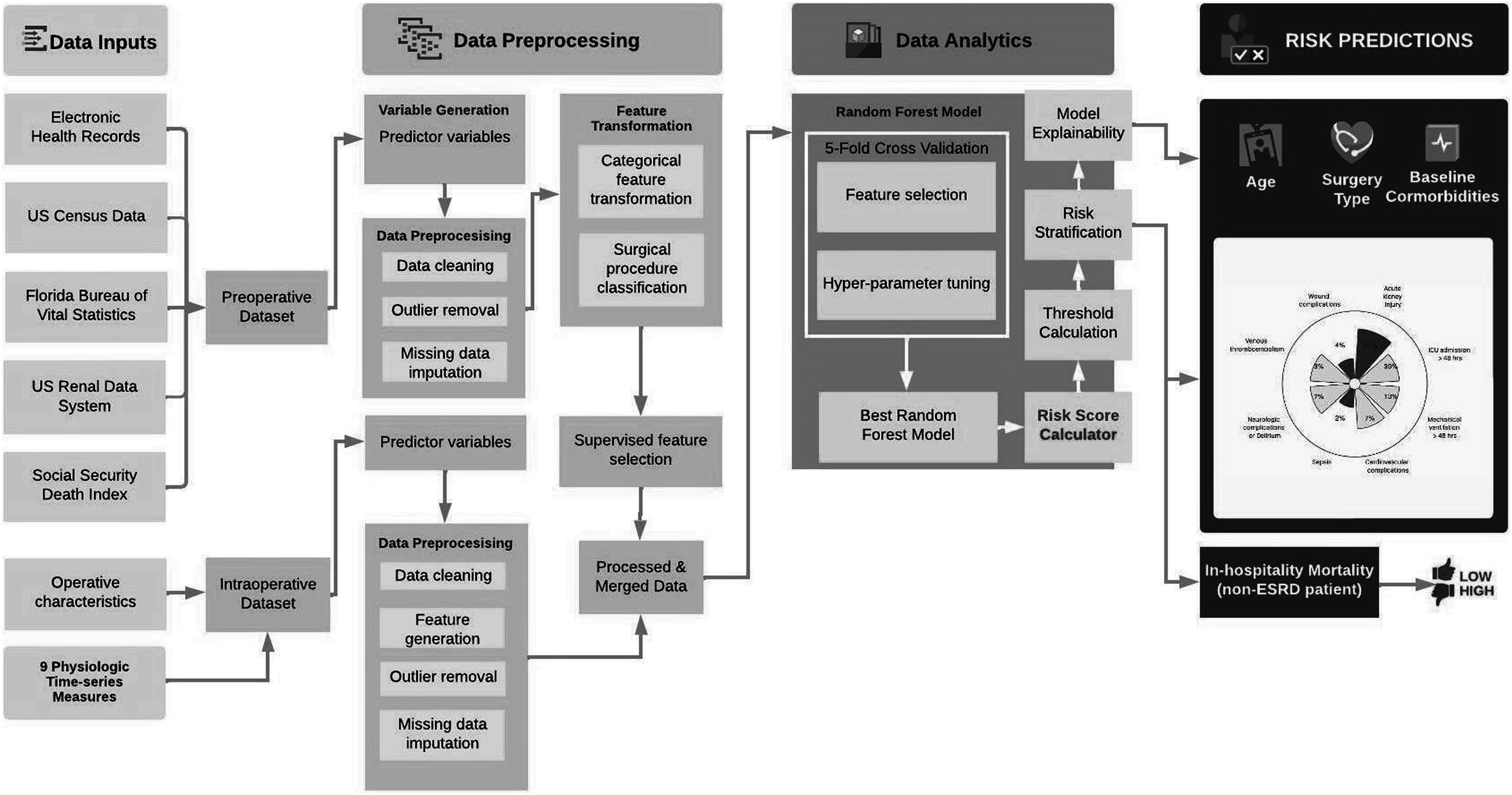
Current model architecture for *MySurgeryRisk* analytics platform. The diagram shows sequence of steps from aggregation of raw data, data engineering and data analytics to final output showing risk estimate.

The model architecture for future designs was described by Feng *et al* (2017). The system was built of three principal components: Data Producer, Intelligent Engine, and Application Client (see figure 1). The first component aggregates data from multiple databases. The Engine is housed under the Docker production environment and hosts a distributed computational infrastructure (SparkSQL), a NoSQL database (Cassandra), and a distributed file solution (HDFS). Predictions were based on generalized additive models. The outputs include a real-time prediction of possible complication, batched model training, and data-base analysis with SQL. The outputs would be made more interpretable by passing them along into web-based application that visualizes the data into colorful graphs (see figure 2).

### My surgery risk

*MySurgeryRisk* was published in 2018 as part of the Precision and Intelligent Systems in Medicine (Prisma^P^) and Intelligent Health Lab (i-Heal) groups at the University of Florida (figure 2) (Bihorac *et al* 2019). The *MySurgeryRisk* algorithm consisted of a Data Transformer and Data Analytics modules. 51 457 patients with 285 available variables were fed to the algorithm. One-fifth of the cohort was held as a validation set in each of the 50-time repeated 5-fold cross validation runs. The probability of each complication during hospitalization after index surgery, were calculated using a GAM with logistic link function, as described by Thottakarra above. We defined the optimal cutoff values that best categorize patients into low and high risk categories by maximizing the Youden index. Mortality, however, was calculated using best of 675 random forest classifiers.

After validation, complication prediction by *MySurgeryRisk* was found to have AUC values ranging from 0.82 (wound complication, 95% CI 0.81–0.84) to 0.94 (need for prolonged mechanical ventilation, 95% CI 0.93–0.94), with 30 day mortality predicted with AUC 0.83 (CI 0.81–0.85). Other metrics of performance include accuracy (0.70–0.85), sensitivity (0.76–0.86), specificity (0.69–0.85), positive predictive value (0.26 to 0.72), and negative predictive value (0.85–0.98). A follow up paper compared physician performance to the model (Brennan *et al* 2019). Using complete patient charts previously unseen by either the model or the physician, the predictive abilities of physicians gave an AUC of 0.47–0.69 compared with 0.73–0.85 for the model. After interaction with model, physicians improved their AUC by approximately 2%–5% across various complications with net reclassification of between 12% and 16%.

The *MySurgeryRisk* platform was applied to a new validation cohort of 19 132 patients from November 2018 to September 2020 (Ren *et al* 2022). Generalized Additive Models were compared with Random Forest Models using 55, 101, and 135 features to assess for best model performance. The random forest model trained with highest number of features resulted in the highest AUC values (0.77–0.91) for 10 post-operative complications. Accuracy, sensitivity, and specificity ranged from 0.64 to 0.84 across all predictions and can be found in the supplemental table.

## Improved performance with intraoperative data

Our group next began to incorporate intra-operative data into our prediction models. Given our interest in acute kidney injury, we first developed a model with specific interest in this complication (Adhikari *et al* 2019). In addition to previously referenced predictor features, we also added five physiologic time series data (mean arterial blood pressure, systolic blood pressure, diastolic blood pressure, minimum alveolar concentration of inhaled anesthetics, and heart rate), 21 repeated laboratory measures, and other variables (intraoperative medications, duration of the operation, anesthesia type). We used the initial 2000–2010 data set to build the model and named the new model IDEA (Intraoperative Data Embedded Analytics). It took previous preoperative risk calculations using the generalized additive model and passed it to a stacked model using a random forest approach. The new model had an AUC of 0.86 (95% CI 0.85–0.90) compared with 0.84 (95% CI 0.82–0.87) in the previous model, with improved sensitivity (from 0.68 to 0.81) and negative predictive value (from 0.79 to 0.85). The authors noted a reclassification of 40% of false negative patients.

Datta *et al* incorporated these processes into the latest version of *MySurgeryRisk*, whose architecture is show in figure 2 (Datta *et al* 2020). Many of our current post-operative risk prediction models rely solely on pre-operative data, ignoring the many physiologic changes that occur intraoperatively. Compared with our AKI work, this model was built using our updated IDR database with 2014–2019 data with more predictor features. This model similarly combined data from both pre- and intra-operative data transformers into a Random Forest Model with 5-fold cross validation. We found a net reclassification index ranging from 0.02 (wound complication) to 0.21 (duration of mechanical ventilation >48 h) with overall classification improvement ranging from 2.4% (wound complication) to 11.2% (hospital mortality).

## DeepSOFA

In 2018, Shickel and colleagues developed our first application of deep learning in generating real-time patient acuity scores (Shickel *et al* 2019). Our model was built after a thorough review of the literature of deep learning techniques for analysis of EHR data (Shickel *et al* 2018). We noted which state-of-art technologies were used for: extraction of free-text, billing codes, and quantitative variables; representation; outcome prediction for both static and temporal data; computational phenotyping for medical personalization; de-identification for universal data sharing; and interpretability to aid in clinical adoption. Methods for evaluating the reliability these technologies were also reviewed. It was noted that most technologies are focused on piecemeal extraction of data types in isolation, as static or temporal forms of billing codes, text, images, vitals, lab values. At the time, the future promise of these technologies was integration of these diverse sets of information into a single database.

**Figure 3.**
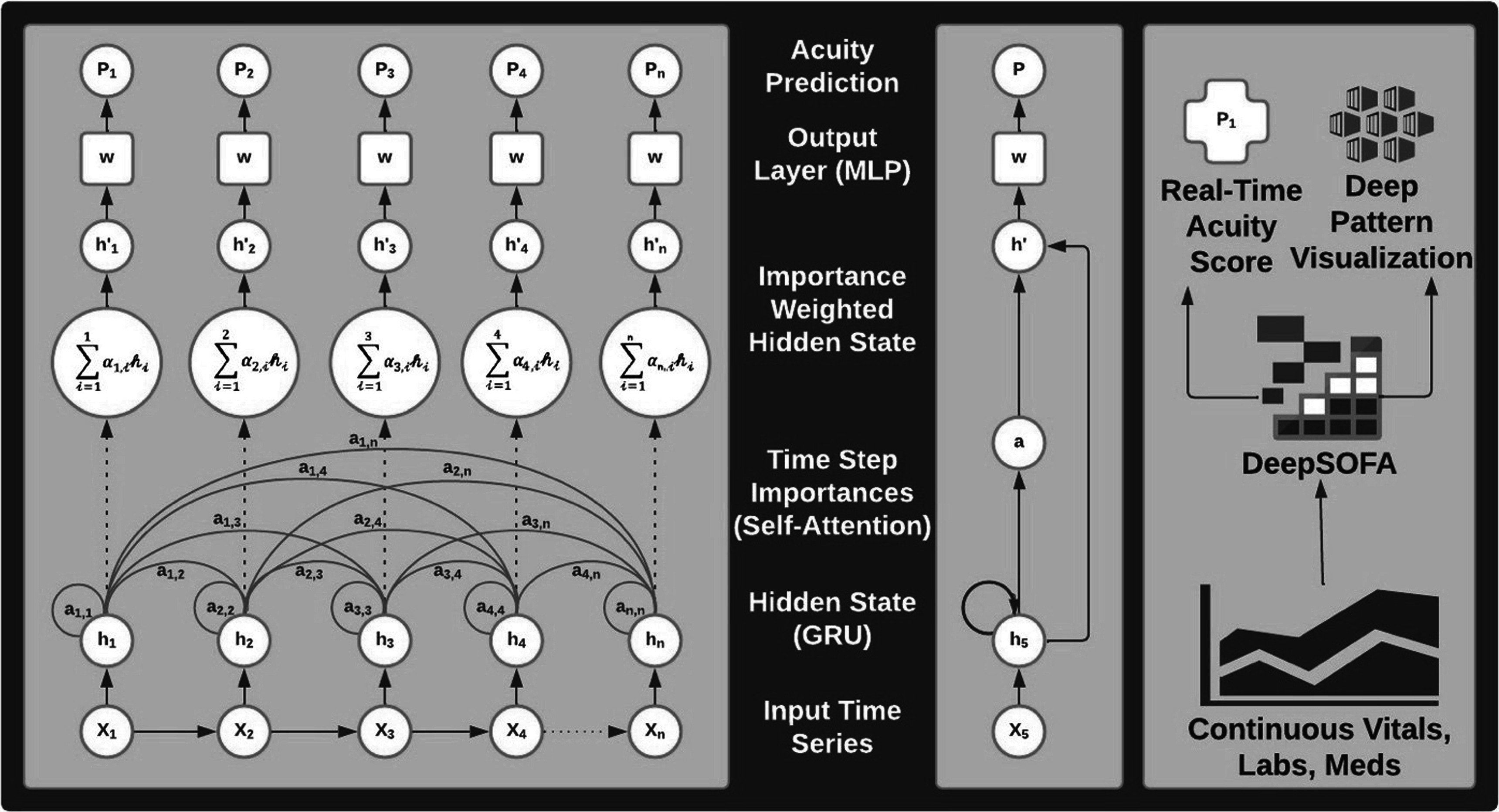
Three views of the DeepSOFA model with increasing levels of abstraction. Panel A shows mortality prediction calculation for a sample patient over a period of *n* hours in the ICU. As time advances, there is communication between the present node and all past nodes, which are passed forward into the neural network. Panel B shows a compact form of each stage while Panel C shows the upper-level acuity assessment and interpretable prediction rationale.

Using this background, we developed a recurrent neural network with gated recurrent units to build DeepSOFA (figure 3), a software capable of extracting EHR data to create real-time predictions of mortality based off traditional SOFA scores (Shickel *et al* 2019). Recurrent Neural Networks are especially suited to learn the structure and relationships among sequential/temporal trends from low-level data. Gated recurrent units ‘decide’ which vectors to pass along and which to forget, allowing for longer retention of previous data. These calculations are performed automatically, while traditional multivariable, static regression models–including traditional and bedside SOFA scores–are both inferior and more cumbersome. The DeepSOFA models resulted in significantly more accurate predictions of in-hospital mortality both for the entire ICU stay (AUC 0.90, 9CI 0.90–0.91 versus 0.88, 95% CI0.88–0.89) and within the first several hours (AUC 0.74, 95% CI0.73–0.75 versus 0.67, 95% CI0.66–0.67).

## Intelligent ICU

Similar efforts have been evaluated in our ICU with expanded physiologic criteria. This includes a particular interest in delirium, a common postoperative complication. In 2018 Malhotra *et al* used Mask R-CNN, a Region-based Convolutional Neural Network optimized for image and instance segmentation, to identify disruptions secondary to staff and family (Malhotra *et al* 2018). This automated imaging system was able to quantify visitations to a patient’s room with the aim of improving visitation polices and optimizing timing for procedures.

In the last few years, we have experimented with more pervasive forms of monitoring. Wearable technologies, light detection, noise meters, and high-resolution cameras can feed data into a central repository to identify early signs of delirium in ICU patients. In a prospective study, 22 patients were recruited from out ICU and studied using a combination of more traditional measures of ICU Delirium (CAM-ICU, DMSS-4, and MDAS) with deep learning (Davoudi *et al* 2019). Variables included physiologic and electronic health record data, but also facial expression recognition, head pose detection, posture recognition, extremity movement analysis, visitation frequency, room sound and light levels, sleep characteristic. We used previously built deep and machine learning models, many of which are detailed in a systematic review by our group (Demrozi *et al* 2020). The Joint Face Detection and Alignment using a Multi-Task Cascaded Convolutional Network allows computers to recognize faces, while the OpenFace deep neural network tool can aide with expressions related to pain, anger, surprise, or happiness (Amos *et al* 2016, Zhang *et al* 2016). Posture was identified with multi-person 2D pose estimation model with part affinity fields, a Fully Convolutional Neural network (Cao *et al* 2017). This proof-of-concept study had several limitations but was able to separate delirious from non-delirious patients across multiple measures, with potential application for reducing disruptions and alleviating nurse workload.

This expanded set of inputs can then be passed to a different set of outputs. We trialed an Intelligent ICU with novel environmental and pervasive sensing technology for 51 patients to assess successful (home or rehabilitation) versus unsuccessful (death or hospice) hospital discharge (Shickel *et al* 2021). We limited this first model to the motion-detecting accelerometer after a review of the literature assessing most important variables for pervasive monitoring in the ICU (Davoudi *et al* 2022). Equally important, this study also sought to evaluate transfer learning, where we trained a RNN on a traditional ICU cohort and used it is discovered weight and biases for our intelligent ICU. The addition of accelerometer data did not appreciably alter AUC for prediction of hospital discharge status based on our single cohort (EHR data AUC 0.73, 95% CI 0.62–0.83; EHR data along with Intelligent data, 0.74, 95% CI0.64–0.84). However, the addition of transfer learning greatly improved both (AUC 0.83, 95% CI0.56–0.95; 0.92, 95% CI0.77–0.98) and suggests the importance of using larger datasets to train new models of smaller cohorts.

## Regulation, ethics, and external validation

As we solidify the technical aspects of model development, our group is now focusing on implementation. The United States Food and Drug Administration regulates prediction models as ‘Software as Medical Device’ (proposed regulatory framework for modifications to artificial intelligence/machine learning AI/ML-based software as a medical device SaMD 2019). As we have yet to apply our model in clinical practice, we have not yet sought FDA approval, though we work closely with our legal and technology transfer offices regarding artificial intelligence regulation.

Predictive models are also fraught with ethical issues. Equity remains of chief concern, and we have the ability to weight unrepresented groups in future iterations of our model to avoid potential bias (Johnson-Mann *et al* 2021). Continuous video monitoring of subjects in our studies were performed with full consent of the patient or their designated healthcare surrogate. We envision the use of video monitoring would fall under the same legal realm as seizure monitoring with electroencephalography when clinically indicated.

For additional testing and validation of our models, we used the Medical Information Mart for Intensive Care (MIMIC), a freely accessible, single-center, database through Beth Israel Deaconess Medical Center (Johnson *et al* 2016). MIMIC and IDEALIST share nearly all relevant datapoints, including demographics, bedside monitoring, diagnostic codes, procedure codes, medication logs, and clinical text. Validation has also been performed on data from our partner hospitals, including the University of Florida Health Jacksonville. The next step in external validation involves federated learning, a machine learning technique that shares model parameters and hyperparameters rather than the underlying variables. This allows improved model training without concern for transfer of patient identifiers. We are working closely with four other academic institutions to validate *MySurgeryRisk* using this technology (Loftus *et al* 2022a).

Finally, medical practice continues to change at a fast pace, and it has been suggested that clinical data decays with a ‘half-life’ of about 4 months (Chen and Asch 2017, Chen *et al* 2017). Our retrospective models are updated as new training data becomes available.

## Future directions

Our work with the *MySurgeryRisk* application continues to grow in several directions.

Most importantly, we are working on integration into clinical workflow. We have designed a mobile web-based application according to user interface principles (Ren *et al* 2022). The application first presents the daily operating room schedule and aggregates data from the EHR for the surgeon’s patient, including basic demographics, medications, recent laboratory values, area development index score, and risk score for eight post-operative complications. As the model also measures intra-operative variables, it will update risk measures to aid in post-operative triage decisions. We have shown how over- and under-triage may harm patients or result in lower value of care using our risk prediction algorithms (Loftus *et al* 2021, 2022b). Pilot usability testing is currently on-going at our institution to develop a decision support tool.

Adoption, however, will also require explainability and we are exploring different regularization techniques to help clinicians understand why a model reached a certain prediction. All our models can show feature importance and the self-attention mechanism impeded into the DeepSOFA algorithm highlight when and what the model found most relevant, however, these are not explicitly displayed in our application. There is a debate about the value added in these explanations and we will explore physician attitudes towards explainability in our usability testing (Ghassemi *et al* 2021).

*MySurgeryRisk* features also continue to expand. We are currently testing the models with ventilator settings, current lines and drains, and patient-centered features, like goals of care, in addition to improving model performance with higher-risk surgeries and patient populations (Ruppert 2022). Finally, the University of Florida houses GatorTron, one of the largest clinical language learning models (Yang *et al* 2022). Incorporation of clinical text into models is a new frontier in risk prediction which we are actively exploring through natural language processing techniques.

## Conclusions

Our group is committed to developing predictive tools for integration of artificial intelligence into the clinical workflow. Our current work focuses on predictive analytics and, as described above, we have applied best practices to the development of the *MySurgeryRisk* calculator and our Intelligent Critical Care Center.

We believe building a real-time model is essential to quantifying surgical risk. These tools continue to improve in both technological sophistication and clinical utility with each iteration.

## Funding

JAB was support by the National Institute of General Medical Sciences (NIGMS) under award number 5T32GM008721–24. TJL was supported by the NIGMS of the National Institutes of Health under Award Number K23GM140268. TOB was supported by K01 DK120784, R01 DK123078, and R01 DK121730 from the National Institute of Diabetes and Digestive and Kidney Diseases (NIH/NIDDK), R01 GM110240 from the National Institute of General Medical Sciences (NIH/NIGMS), R01 EB029699 from the National Institute of Biomedical Imaging and Bioengineering (NIH/NIBIB), R01 NS120924 from the National Institute of Neurological Disorders and Stroke (NIH/NINDS), AGR DTD 12-02-20 from University of Florida Research, and UL1TR001427 from the National Center For Advancing Translational Sciences of the National Institutes of Health. PJT was supported by R01GM114290 from the NIGMS and R01AG121647 from the National Institute on Aging (NIA). PR was supported by National Science Foundation CAREER award 1750192, 1R01EB029699 and 1R21EB027344 from the National Institute of Biomedical Imaging and Bioengineering (NIH/NIBIB), R01GM-110240 from the National Institute of General Medical Science (NIH/NIGMS), 1R01NS120924 from the National Institute of Neurological Disorders and Stroke (NIH/NINDS), and by R01 DK121730 from the National Institute of Diabetes and Digestive and Kidney Diseases (NIH/NIDDK). A.B. was supported R01 GM110240 from the National Institute of General Medical Sciences (NIH/NIGMS), R01 EB029699 and R21 EB027344 from the National Institute of Biomedical Imaging and Bioengineering (NIH/NIBIB), R01 NS120924 from the National Institute of Neurological Disorders and Stroke (NIH/NINDS), and by R01 DK121730 from the National Institute of Diabetes and Digestive and Kidney Diseases (NIH/NIDDK). This work was also supported in part by the NIH/NCATS Clinical and Translational Sciences Award to the University of Florida UL1 TR000064.

## References

[pmeaacb4dbbib1] Adhikari L (2019). Improved predictive models for acute kidney injury with IDEA: intraoperative data embedded analytics. PLoS One.

[pmeaacb4dbbib2] ai.ufl.edu-AI-University of Florida (2022). https://ai.ufl.edu/.

[pmeaacb4dbbib3] Amos B, Ludwiczuk B, Satyanarayanan M (2016). Openface: a general-purpose face recognition library with mobile applications. CMU School Comput. Sci..

[pmeaacb4dbbib4] Bihorac A (2013). National surgical quality improvement program underestimates the risk associated with mild and moderate postoperative acute kidney injury. Crit. Care Med..

[pmeaacb4dbbib5] Bihorac A (2019). MySurgeryrisk: development and validation of a machine-learning risk algorithm for major complications and death after surgery. Ann. Surg..

[pmeaacb4dbbib6] Brennan M (2019). Comparing clinical judgment with the MySurgeryRisk algorithm for preoperative risk assessment: a pilot usability study. Surgery.

[pmeaacb4dbbib7] Cao Z, Simon T, Wei S-E, Sheikh Y (2017). Realtime multi-person 2d pose estimation using part affinity fields.

[pmeaacb4dbbib8] Chen J H, Alagappan M, Goldstein M K, Asch S M, Altman R B (2017). Decaying relevance of clinical data towards future decisions in data-driven inpatient clinical order sets. Int. J. Med. Inform..

[pmeaacb4dbbib9] Chen J H, Asch S M (2017). Machine learning and prediction in medicine—beyond the peak of inflated expectations. New Engl. J. Med..

[pmeaacb4dbbib10] Cho K (2014). Learning phrase representations using RNN encoder–decoder for statistical machine translation. http://arxiv.org/abs/1406.1078.

[pmeaacb4dbbib11] Copelan G P, Jone D, Walter M (1991). POSSUM: a scoring system for surgical audit. Br J Surg..

[pmeaacb4dbbib12] Corey K M (2018). Development and validation of machine learning models to identify high-risk surgical patients using automatically curated electronic health record data (Pythia): a retrospective, single-site study. PLoS Med..

[pmeaacb4dbbib13] Datta S (2020). Added value of intraoperative data for predicting postoperative complications: the mysurgeryrisk postop extension. J. Surg. Res..

[pmeaacb4dbbib14] Davoudi A (2019). Intelligent ICU for autonomous patient monitoring using pervasive sensing and deep learning. Sci. Rep..

[pmeaacb4dbbib15] Davoudi A, Shickel B, Tighe P J, Bihorac A, Rashidi P (2022). Potentials and challenges of pervasive sensing in the intensive care unit. Front. Digit. Health.

[pmeaacb4dbbib16] Demrozi F, Pravadelli G, Bihorac A, Rashidi P (2020). Human activity recognition using inertial, physiological and environmental sensors: a comprehensive survey. IEEE Access.

[pmeaacb4dbbib17] Dencker E E, Bonde A, Troelsen A, Varadarajan K M, Sillesen M (2021). Postoperative complications: an observational study of trends in the United States from 2012 to 2018. BMC Surg..

[pmeaacb4dbbib18] Feng Z (2017). Intelligent perioperative system: towards real-time big data analytics in surgery risk assessment. DASC PICom DATACom CYBERSciTech.

[pmeaacb4dbbib19] Ghassemi M, Oakden-Rayner L, Beam A L (2021). The false hope of current approaches to explainable artificial intelligence in health care. Lancet Digit. Health.

[pmeaacb4dbbib20] Hashimoto D, Meireles O, Rosman G (2021). Artificial Intelligence in Surgery: Understanding the Role of AI in Surgical Practice.

[pmeaacb4dbbib21] Hyder J A, Reznor G, Wakeam E, Nguyen L L, Lipsitz S R, Havens J M (2016). Risk prediction accuracy differs for emergency versus elective cases in the ACS-NSQIP. Ann. Surg..

[pmeaacb4dbbib22] Intelligent Critical Care Center (IC3) (2022). https://ic3.center.ufl.edu.

[pmeaacb4dbbib23] Johnson A E (2016). MIMIC-III, a freely accessible critical care database. Sci. Data.

[pmeaacb4dbbib24] Johnson-Mann C N, Loftus T J, Bihorac A (2021). Equity and artificial intelligence in surgical care. JAMA Surg..

[pmeaacb4dbbib25] Loftus T J (2021). Association of postoperative undertriage to hospital wards with mortality and morbidity. JAMA Netw Open.

[pmeaacb4dbbib26] Loftus T J (2022a). Federated learning for preserving data privacy in collaborative healthcare research. Digit. Health.

[pmeaacb4dbbib27] Loftus T J (2022b). Postoperative overtriage to an intensive care unit is associated with low value of care. Ann. Surg..

[pmeaacb4dbbib28] Malhotra K R, Davoudi A, Siegel S, Bihorac A, Rashidi P (2018). Autonomous detection of disruptions in the intensive care unit using deep mask RCNN.

[pmeaacb4dbbib29] Proposed regulatory framework for modifications to artificial intelligence/machine learning (AI/ML)-based software as a medical device (SaMD) (2019). www.fda.gov:.

[pmeaacb4dbbib30] Protopap K L, Simpso J C, Smit N C, Moonesingh S R (2014). Development and validation of the Surgical Outcome Risk Tool (SORT). Br J Surg.

[pmeaacb4dbbib31] Reilly J R, Gabbe B J, Brown W A, Hodgson C L, Myles P S (2021). Systematic review of perioperative mortality risk prediction models for adults undergoing inpatient non-cardiac surgery. ANZ J. Surg..

[pmeaacb4dbbib32] Ren Y (2022). Performance of a machine learning algorithm using electronic health record data to predict postoperative complications and report on a mobile platform. JAMA Netw. Open.

[pmeaacb4dbbib33] Ruppert M R (2022). Optimization of Inpatient Level of Care Through Multi-Task Learning.

[pmeaacb4dbbib34] Sangji N F (2016). Derivation and validation of a novel Emergency Surgery Acuity Score (ESAS). J Trauma Acute Care Surg..

[pmeaacb4dbbib35] Saria S, Rajani A K, Gould J, Koller D, Penn A A (2010). Integration of early physiological responses predicts later illness severity in preterm infants. Sci. Transl. Med..

[pmeaacb4dbbib36] Shickel B, Davoudi A, Ozrazgat-Baslanti T, Ruppert M, Bihorac A, Rashidi P (2021). Deep multi-modal transfer learning for augmented patient acuity assessment in the intelligent ICU. Front. Digit. Health.

[pmeaacb4dbbib37] Shickel B, Loftus T J, Adhikari L, Ozrazgat-Baslanti T, Bihorac A, Rashidi P (2019). DeepSOFA: a continuous acuity score for critically ill patients using clinically interpretable deep learning. Sci. Rep..

[pmeaacb4dbbib38] Shickel B, Tighe P J, Bihorac A, Rashidi P, Deep E H R (2018). A survey of recent advances in deep learning techniques for electronic health record (EHR) analysis. IEEE J. Biomed. Health Inform..

[pmeaacb4dbbib39] Stanford Health Care: Surgery Statistics: @StanfordHealth (2022). https://stanfordhealthcare.org/medical-clinics/surgery-clinic/patient-resources/surgery-statistics.html.

[pmeaacb4dbbib40] Thottakkara P (2016). Application of machine learning techniques to high-dimensional clinical data to forecast postoperative complications. PLoS One.

[pmeaacb4dbbib41] Tighe P J, Lucas S D, Edwards D A, Boezaart A P, Aytug H, Bihorac A (2012). Use of machine-learning classifiers to predict requests for preoperative acute pain service consultation. Pain Med..

[pmeaacb4dbbib42] Yang X (2022). http://arxiv.org/abs/2203.03540.

[pmeaacb4dbbib43] Zhang K, Zhang Z, Li Z, Qiao Y (2016). Joint face detection and alignment using multitask cascaded convolutional networks. IEEE Signal Process Lett..

